# Morphological and Molecular Characterization of *Eimeria* spp. Infecting Domestic Poultry *Gallus gallus* in Riyadh City, Saudi Arabia

**DOI:** 10.3390/microorganisms11030795

**Published:** 2023-03-21

**Authors:** Mohammed M. Mares, Saleh Al-Quraishy, Rewaida Abdel-Gaber, Mutee Murshed

**Affiliations:** Department of Zoology, College of Science, King Saud University, P.O. Box 2455, Riyadh 11451, Saudi Arabia; mmares@ksu.edu.sa (M.M.M.); al-quraishi@yahoo.com (S.A.-Q.); rabdelgaber.c@ksu.edu.sa (R.A.-G.)

**Keywords:** *Gallus gallus*, coccidiosis, oocysts, nested PCR

## Abstract

Coccidiosis in chickens is one of the major problems in the poultry industry, caused by protozoan parasites of the genus *Eimeria*. The current study used morphological and molecular characteristics to identify *Eimeria* spp. infecting domestic chickens (*Gallus gallus)* in the Riyadh region of Saudi Arabia. In this study, 120 domestic poultry were examined and 30 were found to be infected with oocysts of *Eimeria* spp. (25%). According to the morphology of the recorded oocysts, five species were found. *Eimeria necatrix* was the first species discovered, and it was distinguished by oblong, ovoid-shaped oocysts with double-layered walls that measured 20 (23–23) and 17 (16–20) μm. The second species was *Eimeria maxima*, which had oval- to egg-shaped oocysts with double-layered walls and measurements of 28 (26–29) and 23 (20–24) μm. The third species was *Eimeria tenella*, characterized by oval-shaped oocysts with double-layered walls and measurements of 21 (20–24) × 17 (16–20) μm. *Eimeria praecox* was the fourth species that was characterized by spherical-shaped oocysts with single-layered walls and measurements of 21 (19–23) × 20 (19–20) μm. *Eimeria acervulina* was the last species to have oval-shaped oocysts with double-layered walls and measurements of 20 (18–25) and 17 (14–20) μm. The percentages of infection with *Eimeria* species were as follows: *E. tenella,* 10.84%; *E. necatrix,* 5.84%; *E. acervulina,* 4.16%; *E. maxima,* 2.5%; and *E. praecox,* 1.66%. Nested PCR based on the amplification of internal transcribed spacer I (ITS-I) regions confirmed the presence of the five *Eimeria* species in the examined fecal samples with their specific amplicon sizes: *E. necatrix* (383 bp), *E. maxima* (145 bp), *E. tenella* (278 bp), *E. praecopx* (116 bp), and *E. acervulina* (321 bp).

## 1. Introduction

Poultry plays an important role in meeting human needs for white meat products and eggs rich in animal protein, providing 30% of animal-based protein as well as income and employment generation and raw materials to some industries [1]. Over the last 20 years, the demand for poultry products has increased greatly among consumers, especially broiler meat and eggs, because they are the cheapest animal protein sources; thus, globally, the annual productions of chicken meat and eggs have averaged approximately 90 million tons and 1.1 trillion tons, respectively [2,3]. Poultry is exposed to many different pathogens, just as other living organisms are, and the most famous and important of these pathogens are coccidia parasites.

Coccidiosis is recognized as a parasitic disease of the intestinal tract of poultry caused by apicomplexan parasites of the genus *Eimeria* [1]. This disease attacks many vertebrates such as mammals, birds, reptiles, fish, and amphibians, as well as many invertebrates [2]. The life cycle of coccidiosis is characterized by the presence of the phenomenon of a succession of generations, as it includes three basic stages: the schizogony phase, the gametogenic phase, and the sporogony phase. The *Eimeria* life cycle is approximately 4–7 days, beginning when an active oocyst is ingested and swallowed by a bird. Oocysts are thick-walled capsules that protect the parasites, which become sporulated or infective when moisture, temperature, and oxygen promote their growth. After birds eat the oocysts, coccidia invade the intestinal lining and multiply several times, damaging the tissue [4]. These infections result in great losses in the poultry industry due to poor growth, digestion disability, reductions in egg production, morbidity, and mortality that can be as high as 80% [3]. Therefore, *Eimeria* parasites are of great interest to poultry and livestock breeders because of the losses they cause [4]. Annual global poultry production losses are consequently large and are estimated at billions of dollars [4].

In the poultry industry, chickens can be infected by any of the seven Eimeria species, including *E. tenella*, *E. maxima*, *E. acervulina*, *E. necatrix*, *E. brunette*, *E. mitis*, and *E. praecox*, which cause coccidiosis with variable levels of pathogenicity [5,6]. *E. acervulina* and *E. maxima* are the most prevalent, and *E. tenella* is the most common among the highly pathogenic species. Infection with *E. tenella* can lead to high mortality as a result of moderate to severe damage to the intestinal epithelium due to deep penetration of the cecum tissues, causing cecal subepithelial hemorrhagic lesions [5]. *E. necatrix* produces major lesions in the jejunum and caeca portions of the small intestine. Small white spots, usually intermingled with rounded, bright- or dull-red spots of various sizes, can be seen on the serosal surface. *E. acervulina* is the most common cause of infection. Lesions include numerous whitish, oval, or transverse patches in the duodenum and jejunum of the small intestine. *E maxima* develop in the duodenum, jejunum, and ileum, where they cause dilatation and thickening of the wall, petechial hemorrhage, and a reddish, orange, or pink viscous mucous exudate and fluid. The exterior of the midgut often has numerous whitish pinpoint foci, and the area may appear engorged. *E. praecox*, which infects the duodenum and jejunum, does not cause distinct lesions but may decrease the rate of growth. The intestinal contents may be watery. *E. praecox* is considered to be of less economic importance than other species [7,8]. In poultry, the *Eimeria* parasites cause an imbalanced status of oxidants/antioxidants [6], and thus, oxidative stress occurs.

The intestinal epithelial barrier and tight junctions are destroyed through lipid peroxidation and antioxidant insult, resulting in reduced feed intake, poor absorption of nutrients, and decreased body weight gain (BWG) in the infected birds [7]. The diagnosis of infection with *Eimeria* parasites is determined through the symptoms that appear in poultry in terms of the presence of bloody diarrhea in the stool [9], the presence of oocysts of different shapes in the feces, and changes that occur in the location of the infection in the intestinal mucosa [10,11]. The distribution and spread of poultry coccidiosis are affected by many factors, including the high density of birds in a small area, the ambient air temperature, high humidity, different ages of birds in the same place, the quality of feed, and the general health status of birds. The severity of the disease depends on the age of the birds and their ability to respond with immunity to the disease [12,13].

The identification and genetic characterization of different species of *Eimeria* are vital for the prevention, surveillance, and control of coccidiosis. These characterizations are particularly important considering the appearance of widespread anticoccidial resistance in *Eimeria* species and the problems associated with drug residues [14,15]. The most popular methods for identifying these parasites involve observing the oocyst morphology, the prepatent period, the site of infection, or the minimum sporulation time [10]. The results of these findings could be useful for future coccidiosis vaccine development and serve as a reference for effective chemoprevention and control strategies [12,16]. However, these conventional approaches are time-consuming, and the overlap between variable morphological and biological features of different species limits identification accuracy in field conditions with mixed infections [17].

Recently, polymerase chain reaction (PCR) based on the amplification of DNA has been used for the accurate identification of *Eimeria* species involved in single or mixed infections. PCR assays have gained popularity over traditional methods due to their reliance on the genomic sequence of *Eimeria* species [18,19].

The current study aimed to combine morphological and molecular methods so as to identify the various *Eimeria* species found in domestic chickens in Riyadh, Saudi Arabia.

## 2. Materials and Methods

### 2.1. Design and Collection of Study Animals

A total of 120 domestic chickens (*Gallus gallus*) were purchased from a local market in Riyadh, Saudi Arabia. All 120 chickens were collected randomly in batches over a period of 6 months, with each batch containing 40 chickens. The chickens were examined to calculate the incidence rate and number of chickens non-infected with coccidiosis. Each chicken was individually housed in wire-floored batteries under sanitary conditions in the animal house at the Zoology Department of the College of Science at King Saud University. Water and feed were provided to the poultry ad libitum.

### 2.2. Detection of Coccidiosis Natural Infection

Every 24 h, feces were collected individually from each chicken. There were special cages for each chicken, with dishes under each cage, from which we collected the chicken feces. Thus, the chicken feces were collected separately. Then, a total of 2 g of each sample was placed in 60 mL of saturated saline. The suspension was filtered through cheesecloth and centrifuged at 1500 rpm for 10 min. A drop of the sample was used to prepare the liquid film for examination under a light microscope. Oocyst-positive samples were exposed to the flotation technique, and oocysts were collected for incubation in 2.5% (*w*/*v*) potassium dichromate and sporulation at 27 °C for 5 days [20]. The sporulated oocytes were stored at 4 °C for molecular studies.

### 2.3. Microscopic Examination

The oocysts of each sample were photographed, and the morphometric characteristics were examined using an Olympus microscope equipped with a digital camera (Olympus, CX41, Tokyo, Japan). The data were expressed as the mean (range).

### 2.4. DNA Extraction

The oocysts were washed with distilled water to remove the potassium dichromate. The washed oocysts were vortexed vigorously with 0.5 mm diameter glass beads to rupture the oocyst wall. DNA was then extracted using the ISOLATE II Fecal DNA Kit (BIOLINE, London, UK) following the manufacturer’s protocol with minor modifications.

### 2.5. Molecular Methods

Nested PCR based on the amplification of internal transcribed spacer I (ITS-I) regions of DNA was standardized using specific primers from *Eimeria*, as recommended by Amer et al. [21]. PCR was performed in a final volume of 20 µL containing 10 µL of PCR master mix, 1 µL of forward primers, 1 µL of reverse primers, 1 µL of DNA template, and enough nuclease-free water to complete the volume of 20 µL. After an initial denaturing temperature of 94 °C for 3 min, 35 cycles of 98 °C for 30 s, 56 °C for 30 s, and 72 °C for 1 min were run, followed by a final extension at 72 °C for 5 min. All PCR products were analyzed by first separating 5 μL of product on 2% agarose gel in TAE buffer at 100 V for 30–45 min, followed by staining with ethidium bromide and examination under a UV light to visualize the expected product size in a gel documentation system. After obtaining the product from the Nested PCR based on the amplification of internal transcribed spacer I (ITS-I) regions, 1 µL of DNA template was taken and the reaction was completed in the same way, except for the change in the annealing temperature, determined according to the type of *Eimeria* (Table 1).

### 2.6. Statistical Analysis

The Sigma Plot program was used (version 11, Systat Software, Inc., San Jose, CA, USA). Duncan’s test was used to compare each treatment on its own, and a one-way analysis of variance was used to establish each treatment’s overall influence on the patient. The values are reported as the mean and the standard deviation, and a *p*-value less than 0.05 was considered statistically significant.

## 3. Results

**Figure 1:**
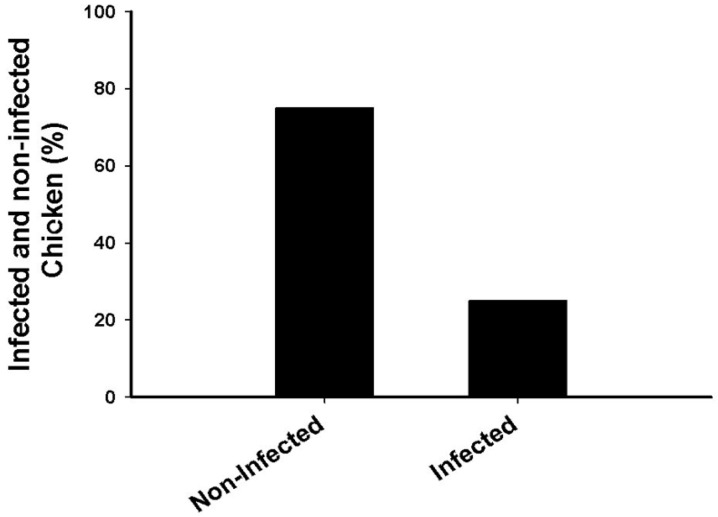
The infection rate of *Eimeria* parasites in domestic chickens.

In the current study, 120 chickens were examined for coccidiosis infection, and 30 chickens were found to be infected with different species of the *Eimeria* genus constituting 25% of the total. The infections were normal, and the chickens were not given any vaccines or treatments against *Eimeria* by the breeders (Figure 1). The total incidence percentages of infection with *Eimeria* species were *E. tenella* 10.84%, *E. necatrix* 5.84%, *E. acervulina* 4.16%, *E. maxima* 2.5%, and *E. praecox* 1.66% for the total 120 chickens, respectively (Table 2).

### 3.1. Morphological Identification

Based on the morphometric data, five *Eimeria* species were detected in the feces of the examined chickens and identified as follows below.

#### 3.1.1. *Eimeria tenella*

**Figure 2:**
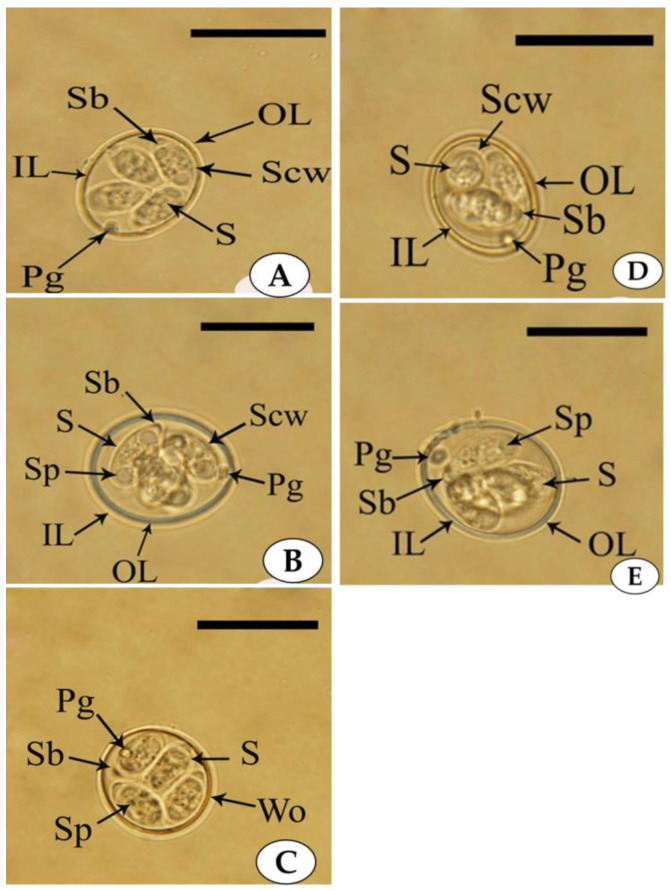
Photomicroscope images showing the sporulated oocysts of *E. maxima* (**A**), *E. tenella* (**B**), *E. acervulina* (**C**), *E. praecox* (**D**), and *E. necatrix.* (**E**) The outer (OL) and the inner (IL) layers of the oocyst wall, as well as the polar granule (Pg), sporocyst (S), Stieda body (Sb), sporocyst wall (Scw), sporozoite (SP), showing the presence of four sporocysts containing two sporozoites in each sporocyst (scale per = 20 µm).

The oocysts are broad and ovoid with double-layered oocyst walls. Micropyle and micropylar caps are absent. The dimensions of the sporulated oocysts are 21 (20–24) μm in length and 17 (16–20) μm in width. A prominent polar granule is present at the anterior end close to the oocyst wall, while the oocyst residuum is absent. The sporocysts are broad, elongated, and slightly tapered at the anterior end, measuring ~10 (10–11) μm in length and 5.8 (5–6) μm in width (Figure 2A).

#### 3.1.2. *Eimeria maxima*

The oocysts are oval- to egg-shaped without micropyle and micropylar caps and with double-layered oocyst walls. The sporulated oocyst shows the presence of a prominent polar granule at the anterior end close to the oocyst wall, and the oocyst residuum is absent. Each oocyst measures 28 (26–29) μm in length and 23 (20–24) μm in width. The sporocyst is elongated, oval, elevated from the front end and measures ~12 (10–12.9) μm in length and 6 (5–7) μm in width (Figure 2B).

#### 3.1.3. *Eimeria praecox*

The oocysts are oval to spherical with a single-layer, thick oocyst wall. Micropyle and micropylar cap are absent. A polar granule is present close to the oocyst wall while the oocyst residuum is absent. The oocyst measurements are 21 (19–23) μm in length and 20 (19–20) μm in width. The sporocysts are elongated, semi-ovoid, and measure ~11 (10–12) μm in length and 6 (5–6) µm in width. The anterior end of the sporocyst is tapered and pointed with a Stieda body, while its the posterior end is rounded (Figure 2C).

#### 3.1.4. *Eimeria acervulina*

The oocysts of this species possess ellipsoidal, double-layered walls without micropyle and micropylar caps. A prominent polar granule is present at the anterior end close to the oocyst wall, and oocyst residuum is absent. The oocyst measurements are 20 (18–25) μm in length and 17 (14–20) μm in width. The sporocysts are typically elongated to ovoid and measure ~12 (8–14) μm in length and 6 (4–6) μm in width. The posterior end of the sporocyst is broad and rounded, while the anterior end is narrow, and a Stieda body is present on its tip (Figure 2D).

#### 3.1.5. *Eimeria necatrix*

The oocysts are oblong and ovoid in shape and covered with double-layered walls. Micropyle and micropylar caps are absent. A prominent polar granule is present at the anterior end just behind the oocyst wall, and the oocyst residuum is absent. The oocyst measurements are 20 (20–23) μm in length and 17 (16–20) μm in width. The sporocysts are typically pyriform in shape and measure ~9 (8–10) μm in length and 5 (4–6) μm in width. The posterior end of the sporocyst is rounded and broad, while the anterior end is narrow and tapered with a large Stieda body (Figure 2E).

### 3.2. Molecular Identification

**Figure 3:**
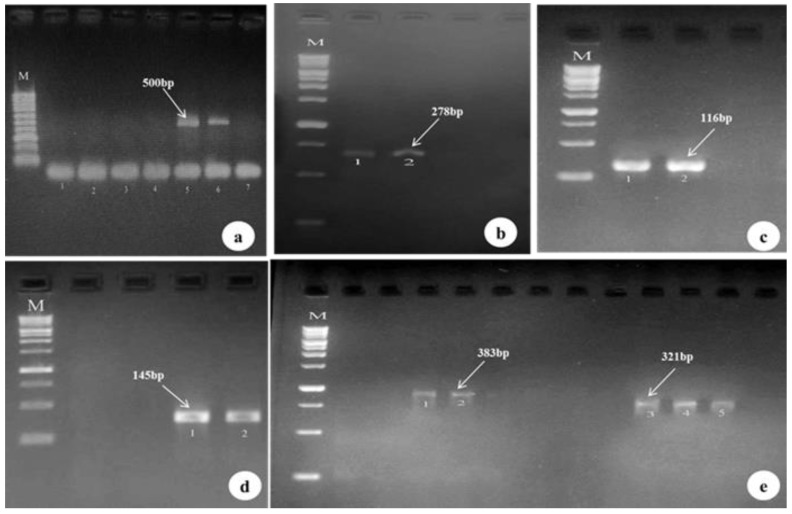
Results were obtained by PCR followed by 2% agarose gel electrophoresis. Lines in M show a 1000 bp DNA marker. Samples include the genus *Eimeria*, 500 bp (**a**), *E. tenella,* 278 bp (**b**), *E. praecox,* 116 bp (**c**), *E. maxima,* 145 bp (**d**), *E. acervulina*, 383 bp and *E. necatrix,* 321 bp (**e**).

A PCR product of 500 bp was successfully amplified from the ITS1 regions of the DNA extracted from the sporulated oocysts. The results of nested PCR revealed the presence of five *Eimeria* species in the examined domestic chickens’ fecal samples. The primers were sufficiently sensitive and specific to enable the discrimination of the five *Eimeria* species. The amplified fragments exhibited different sizes: *E. tenella* (278 bp), *E. praecox* (116 bp), *E. maxima* (145 bp) *E. acervulina* (383 bp), and *E. necatrix* (321 bp) (Figure 3).

## 4. Discussion

Poultry is considered one of the most important components of livestock and a major source of white meat in the Arabian Peninsula and particularly in the Kingdom of Saudi Arabia in particular. However, like other animals, poultry may be infected with some diseases that reduce their productivity. Coccidiosis is one of the most important diseases that affect chickens [22]. In view of the pathological importance of *Eimeria* parasites, some studies have been conducted to survey *Eimeria* in chickens in the Kingdom Saudi Arabia [23]. The current study revealed that chickens in the city of Riyadh, Saudi Arabia were infected with many of these parasites, including *E. tenella*, *E.a maximum*, *E. praecox*, *E. acervulina*, and *E. necatrix*.

The morphological characteristics and measurements of oocysts detected in the present study were compared with those described in other studies, as shown in (Table 3). Even though the majority of the compared species were relatively similar, the morphometric characteristics were slightly different, as follows: The oocysts of *E. tenella* described in the current study were found to be extremely similar to those described by [21]; however, they were smaller in length and wider in width than those described in [24]. *E. tenella* was described morphologically in Sudan, and there was similarity with the species obtained in this study [25]. The oocysts of *E. necatrix* reported in this study were longer than those described by [24,26], although they were similar in breadth to those described by [21]. The *E. acervulina* oocysts in the present study were very similar to those reported by [27] and [21], while they were smaller than those described by [26]. The morphological descriptions of some species of *Eimeria* infecting chickens, including *E. tenella*, *E. necatrix*, *E. acervulina,* and *E. praecox*, in [21] are similar to the morphological characteristics of the oocytes in the current study [21].

The morphometrics of *E. praecox* oocysts in the present study were very similar to those reported by [21,24,26]. Similarly, the oocysts of *E. maxima* were very close to those reported in [30] but smaller than those reported in [27]. The slight variations in oocyst measurements may be attributed to changes in the metabolisms of the parasites or birds and even overlaps in the shape morphometric indices, resulting in misleading conclusions regarding the species [31]. It has been observed that the morphological shapes of the oocysts and sporocysts and their sizes may also change due to changes in the metabolisms of parasites or birds, and these differences may lead to incorrect results in identifying and distinguishing between the types of *Eimeria*. The overlaps between many of these *Eimeria* species represent a problem for morphological characterization [32]. In the Kingdom Saudi Arabia, two new types of *Eimeria*, *E. jeddahnsis* and *E. waeli*, were found to infect chickens in the city of Riyadh. No similarity was observed between these types and the ones described in this study, but this finding is not confirmed and requires a molecular description [33]. In India, three new types of *Eimeria* which infect poultry were described, namely, *E.nikamae, E.tarabaie*, and *E. shivpuri*. There was a difference between the morphological form reported in India and that of the species in this study, but these types require molecular study as it was noted that all new types of *Eimeria* were only described morphological and were not described molecularly. Therefore, molecular study is important for confirming differences between species [32].

Molecular methods have been proven to be a very useful approach for the accurate identification of *Eimeria* species infecting chickens in different geographical regions [34,35]. The ITS1 marker is characterized by a high variability and specific characteristics, such as the AT contents of different groups of organisms, which provide more phylogenetically informative sites. The use of this marker with nested species-specific primers of *Eimeria* in the present study was therefore advantageous in resolving the identity of *Eimeria* spp. and in confirming the morphological descriptions. Consequently, the application of molecular tools for the identification and characterization of these parasites has been carried out to ensure the isolation of five *Eimeria* spp. in Riyadh City, Saudi Arabia by PCR.

The results of the molecular diagnosis, obtained by conventional PCR using the amplified region (ITS-I) marker amplicon of 500 bp with nested species-specific primers of *Eimeria*, proved the presence of five *Eimeria* species in the examined fecal samples of domestic chickens, with their specific amplicon sizes: *E. tenella,* 278 bp; *E. praecox,* 116 bp; *E. maxima,* 145 bp; *E. acervulina,* 383 bp; and *E. necatrix,* 321 bp. The PCR findings coincide with the morphological findings of domestic chicken *Eimeria* species. This study agrees with previous findings reported in [30,36,37,38,39]. In Bangladesh, seven species of *Eimeria* were described in chickens using a PCR test, and it was found that in the cases of *E. tenella*, *E. necatrix*, *E. acervulina*, *E. maxima*, *E. brunetti*, *E. mitis*, and *E. praecox*, there were similarities with the current study findings regarding E. maxima and E. praecox [40]. In China, five types of *Eimeria* were identified in chickens, namely *E. brunetti*, with an the infection rate of 65%, *E. maxima* at 50%, *E. necatrix* at 50%, *E. tenella* at 37%, and *E. acervulina* at 25%. These species were identified by their morphological characteristics and confirmed using PCR [33]. Seven types of *Eimeria* were also identified by PCR examination in Egypt, including *E. praecox*, *E. tenella*, *E. maxima*, *E. mitis*, *E. necatricx*, *E. acervulina*, and *E. brunetti* [41]. Seven types of Eimeria were identified through scar multiplex PCR, including *E. tenella*, *E. necatricx*, *E. acervulina*, *E. brunette*, *E. mitis*, *E. maxima*, and *E. praecox* [42]. These studies agree with the current study, indicating the presence of the same types of *Eimeria* found in different regions of the world, but the methods of diagnosis differed depending on whether morphological or molecular characterizations were applied. *E. tenella* is the predominant *Eimeria* spp. in domestic fowl in Saudi Arabia and worldwide. *E. tenella* is also one of the most common pathogenic causes of clinical symptoms in poultry. Thus, it is necessary to adopt appropriate prophylaxis strategies so as to avoid economic loss. Understanding the occurrence of *Eimeria* species in specific regions using molecular detection methods is useful for choosing an appropriate vaccine to prevent coccidiosis [43,44,45].

The present study provided evidence for the prevalence of *Eimeria* species in poultry based on ITS1-PCR in Riyadh, Saudi Arabia. The presence of *Eimeria* spp. in the study area indicated that the clinical severity of coccidiosis is economically damaging. Most importantly, vaccine development can focus on the most common strains in an effort to control the disease. The detection or specific diagnosis of coccidiosis in chickens by traditional techniques is not reliable enough; therefore, ITS1-based PCR can be recommended for the reliable diagnosis of poultry coccidiosis in Saudi Arabia.

## 5. Conclusions

The present study confirms the presence of *E. tenella*, *E. acervulina*, *E. praecox*, *E. necatrix*, and *E. maxima* circulating in domestic chickens in Riyadh, Saudi Arabia, based on morphological characteristics and confirmed by molecular PCR. The traditional technique for diagnosing coccidiosis in chickens is not sufficiently reliable, but PCR-based methods for DNA sequencing could solve this problem. Furthermore, molecular diagnosis can accurately reveal the prevalence of *Eimeria* species, thus contributing to the strategic employment of medicines and vaccines. Molecular techniques such as multiplex PCR can be improved to rapidly and accurately diagnose *Eimeria* species in field samples.

## Figures and Tables

**Table 1:** Genus-specific and species-specific primers of Eimeria.

Parasite	Primer Sequence 5′ → 3′	Product Size	Annealing Temperature °C
*Genus Eimeria*	EF1: AAGTTGCGTAAATAGAGCCCTC ER1: AGACATCCATTGCTGAAAG	400–600 bp	56
*Eimeria acervulina* (AC)	EAF: GGCTTGGATGATGTTTGCTG EAR: CGAACGCAATAACACACGCT	321 bp	72
*Eimeria praecox* (PR)	EPFA: AAAA/GCAA/CAGCGATTCAAG EPRA: CCAAGCGATTTCATCATT/CGGGGA/G	116 bp	61
*Eimeria maxima* (MA)	EMFA1: CT/ACACCACTCACAATGAGGCAC EMR1: GTGAT/ATCGTTC/TGG/AG/AAGTTTGC	145 bp	70
*Eimeia necatrix* (NE)	ENF: TACATCCCAATCTTTGAATCG ENR: GGCATACTAGCTTCGAGCAAC	383 bp	61
*Eimeria tenella* (TE)	ETF: AATTTAGTCCATCGCAACCCT ETR: CGAGCGCTCTGCATACGACA	278 bp	65

**Table 2:** The percentages of different types of *Eimeria* species in this study.

*Eimeria* Species	NO. Chickens	Infected %
*E. tenella*	13	10.84
*E. necatrix*	7	5.84
*E. acervulina*	5	4.16
*E. maxima*	3	2.5
*E. praecopx*	2	1.66

**Table 3:** Morphological comparison between coccidian oocysts detected in chickens in the present study and other related species from the same host.

Species	Oocyst				Sporocyst		Source
	Measurements	M	MC	PG	SB	SR	
*Eimeria praecox*	19–25 ×15.1–18.7	−	−	+	+	−	[28]
	23.69 × 19.205	−	−	+	+	−	[21]
	19.7–25.7 × 15.6–19.7	−	−	+	+	−	[26]
*Eimeria tenella*	20.0–26.5 ×17.0–22.0	−	−	+	+	−	[29]
	21.39 × 18.745	−	−	+	+	−	[21]
	15–19 × 20–25	−	−	+	+	−	[24]
*Eimeria maxima*	21.5–40.2 × 16.0–29.3	−	−	+	+	−	[27]
	29.9 × 23.8	−	−	+	+	−	[30]
	21.4–42.5 × 16.5–29.8	−	−	+	+	−	[26]
*Eimeria acervulina*	17.3–20.2 × 13.7–16.3	−	−	+	+	−	[26]
	21.96 × 17.48	−	−	+	+	−	[21]
	18.3–27.54 × 13.7–20.40	−	−	+	+	−	[27]
*Eimeria necatrix*	13.2–22.5 × 10.0–18.7	−	−	+	+	−	[28]
	21.27 × 17.71	−	−	+	+	−	[21]
	13.7–22.7 × 11.3–18.3	−	−	+	+	−	[26]

M = micropyle; MC = micropylar cap; PG = polar granule; SB = Stieda body; SR = sporocyst residuum. + = present; − = absent.
